# A comprehensive analysis of sialolith proteins and the clinical implications

**DOI:** 10.1186/s12014-020-09275-w

**Published:** 2020-03-31

**Authors:** Carlos S. Busso, Jessie J. Guidry, Jhanis J. Gonzalez, Vassilia Zorba, Leslie S. Son, Peter J. Winsauer, Rohan R. Walvekar

**Affiliations:** 1 Department of Otolaryngology and Bio-communication, Louisiana State University Medical School Health Sciences Center, 533 Bolivar St. Suite 566, New Orleans, LA 70112 USA; 2 Department of Biochemistry and Molecular Biology, and The LSUHSC Proteomics Facility Core, Louisiana State University Medical School Health Sciences Center, 533 Bolivar St. Suite 331, New Orleans, LA 70112 USA; 3 Laser Technologies Group Energy Storage & Distributed Resources Division, Lawrence Berkeley National Laboratory 70R0108B, University of California Berkeley, 1 Cyclotron Road, Berkeley, CA 94720 USA; 4 Applied Spectra, Inc, 950 Riverside Parkway, West Sacramento, CA 95605 USA; 5 Department of Academic Affairs, Our Lady of the Lake Regional Medical Center, 7777 Hennessy Blvd, Baton Rouge, LA 70808 USA; 6 Department of Pharmacology and Experimental Therapeutics, LSU Health Sciences Center, 1901 Perdido Street, New Orleans, LA 70112 USA; 7 Department of Otolaryngology Head Neck Surgery, Louisiana State University Medical School Health Sciences Center, 533 Bolivar St. Suite 566, New Orleans, LA 70112 USA

**Keywords:** Sialolithiasis, Sialolith, Protein profiling, Extracellular exosomes

## Abstract

**Background:**

Sialolithiasis or salivary gland stones are associated with high clinical morbidity. The advances in the treatment of sialolithiasis has been limited, however, by our understanding of their composition. More specifically, there is little information regarding the formation and composition of the protein matrix, the role of mineralogical deposition, or the contributions of cell epithelium and secretions from the salivary glands. A better understanding of these stone characteristics could pave the way for future non-invasive treatment strategies.

**Methods:**

Twenty-nine high-quality ductal stone samples were analyzed. The preparation included successive washings to avoid contamination from saliva and blood. The sialoliths were macerated in liquid nitrogen and the maceration was subjected to a sequential, four-step, protein extraction. The four fractions were pooled together, and a standardized aliquot was subjected to tandem liquid chromatography mass spectrometry (LCMS). The data output was subjected to a basic descriptive statistical analysis for parametric confirmation and a subsequent G.O.-KEGG data base functional analysis and classification for biological interpretation.

**Results:**

The LC–MS output detected 6934 proteins, 824 of which were unique for individual stones. An example of our sialolith protein data is available via ProteomeXchange with the identifier PXD012422. More important, the sialoliths averaged 53% homology with bone-forming proteins that served as a standard comparison, which favorably compared with 62% homology identified among all sialolith sample proteins. The non-homologous protein fraction had a highly variable protein identity. The G.O.-KEGG functional analysis indicated that extracellular exosomes are a primary cellular component in sialolithiasis. Light and electron microscopy also confirmed the presence of exosomal-like features and the presence of intracellular microcrystals.

**Conclusion:**

Sialolith formation presents similarities with the hyperoxaluria that forms kidney stones, which suggests the possibility of a common origin. Further verification of a common origin could fundamentally change the way in which lithiasis is studied and treated.

## Background

A variety of anomalous stones or calculi occur with a relatively high frequency in certain organs. These formations give rise to a medical condition termed “lithiasis”. The stones predominantly manifest in organs such as kidney (nephrolithiasis), bladder (cystolithiasis), gallbladder (cholelithiasis), bile duct (choledocholithiasis), and salivary glands (sialolithiasis). Lithiasis commonly leads to obstructive or inflammatory effects within these organs and can ultimately decrease organ function. They also cause a high degree of clinical morbidity (pain, swelling, recurrent infections, and organ dysfunction), which may vary depending on the organ affected and the location, number, and invasiveness of stone formation.

According to reports from 2015, in the US alone, the epidemiological incidence of lithiasis reached 23,750 cases/100,000 individuals, 27% of which were treated surgically (L1, and L2).^1^^,^^2^ Based upon very conservative estimations, surgery alone could generate a burden to the healthcare system of around $270 billion. In comparison, sialolithiasis has an incidence of 450 cases per 100,000 individuals/year (mostly treated surgically) (cf. L2, L3 and L4).^3^^,^^4^ This implies that the 3250 cases receiving treatment generate costs of approximately $65 million to the healthcare system. In addition to the economic burden, traditional surgical options may require gland removal and expose the patient to post-operative effects such as cranial nerve injury, xerostomia, and other risks associated with open surgical management (cf. L3, L4, L5, and L6).^5^^,^^6^ Even taking into consideration the newer technologies such as salivary endoscopy, which have reduced the need for gland removal by facilitating stone fragmentation or endoscopic removal, the unpredictable nature of salivary stones in terms of hardness and invasiveness still poses difficulties for successful stone management. The goal of this work, therefore, was to improve means of facilitating lithotripsy or stone dissolution by expanding our knowledge of stone composition; specifically, the role that the protein matrix plays in stone formation and its relation to the constitutive organic and inorganic fractions. In addition, we wanted to explore new methodologies for examining these fractions and comparing them with stone formations in other organs.

Thus, sialolith samples were analyzed using classical population genetic principles [1, 2] and Systems Biology analyses (*ed*: by Rigoustsos and Stephanopoulos [3]) to create specific algorithms designed for addressing our research objectives. Using these algorithms, we were able to investigate unknowns such as the intrinsic variability of salivary stones, the role that the protein matrix plays in the structure and evolution of the stones, and the nature of their interaction with the inorganic phase of the stones. These robust genetic tools were also supplemented using bio-functional classification systems to provide the essential framework for comparisons.

Although our research focused on the protein matrix, we also discuss preliminary data concerning the mineralogical composition and distribution of the inorganic phase. In collaboration with Lawrence Berkeley National Laboratory and Apply Spectra Inc., we have developed a new methodology for analyzing the mineralogical composition of sialoliths using Chemical Imaging.

## Materials and methods

All buffers and compounds employed for sialolith collection, protein extraction, and storage were supplied by Millipore-Sigma and its subsidiaries (St. Louis, MO). Standard laboratory instrumentation used in the project was purchased from Avantor (Allentown, PA) and affiliate corporations of Thermo Fisher Scientific (Waltham, MA). Reagents and instrumentation from Bio-Rad Laboratories (Hercules, CA) were used for protein testing and quantification including gel electrophoresis.

### Patients and samples

The study material was comprised of twenty-nine stones acquired from de-identified patients and they were obtained by the senior author (R.W.) during a surgical procedure to remove them at our study site (Our Lady of The Lake Regional Medical Center, Head and Neck Clinic, Baton Rouge, LA). Informed consent was always granted prior to the surgery.

### Experimental design

**Fig. 1:**
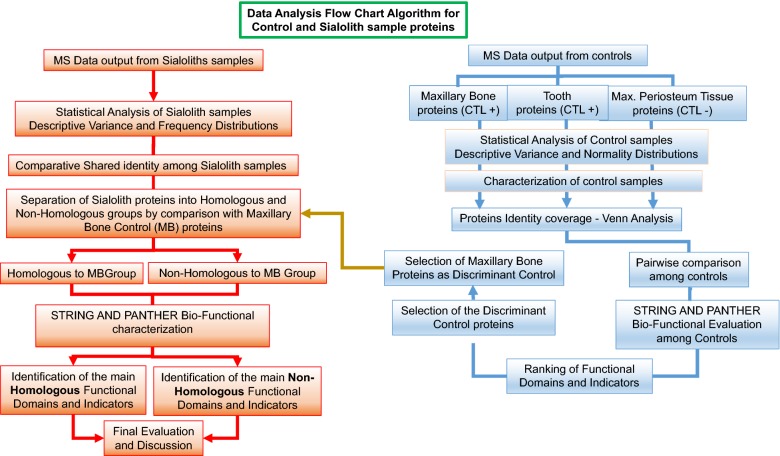
Procedural algorithm flow chart

A flow chart for the design is shown Fig. 1. As indicated, a critical first step in the design was the identification of appropriate comparisons for our study material. Maxillary bone (MB) and tooth (Tt) served as positive comparisons (controls) for homeostatic functional inorganic formations, whereas maxillary periosteal tissue (PT) served as a control for the homeostatic absence of inorganic deposition. In addition, protein identification and characterization parameters from the Mass Spectrometer data output, such as Posterior Error Probability (PEP) and the number of Peptide Spectrum Matches (PSM), were selected for evaluating data quality. The calculated isoelectric point (pI) was our estimator for total protein coverage.

### Surgical isolation of sialoliths

Stone removal was approached using a combination of endoscopic and open techniques. Salivary endoscopes (ranging from 1.1 to 1.6 mm in diameter) with interventional channels that allowed for the insertion of stone capturing baskets were introduced into the salivary ducts after adequate dilation of the ductal orifices in the mouth. Stones were then isolated visually or captured within the stone basket. Stone removal was facilitated by endoscopic extraction or using an additional trans-oral incision to deliver the stones. The stones were immediately placed on gauze dampened with de-ionized water and transferred to a pathology laboratory near the operating room. The stones were code-de-identified before further storage and analysis.

### Sialolith collection and preparation

Stones were washed twice in distilled water, followed by an incubation in a solution of 0.5 M HEPES, 0.05% Triton X-100, and 0.1% SDS to remove external blood and cellular contaminants. The stones were washed twice in a 0.5 M HEPES solution and transferred to a solution consisting of 0.5 M HEPES and ampicillin sodium salt (A9518-25G, Sigma-Aldrich, St. Louis, MO) to eliminate bacterial organisms. The tubes were maintained at 4 ℃ before the protein extraction.

### Protein extraction procedure

Many protein extraction techniques commonly used in the literature were reviewed and studied. Based on this review, a modification of the bone extraction protein method developed by Xiaogang et al. [4] was used for our bone proteomic analysis. This extraction procedure has four sequential steps: (1) the maceration of the stones with liquid nitrogen, (2) demineralization, and (3) two consecutive treatments of the pelleted macerates with guanidine and RIPA buffers, respectively, and (4) total dissolution of the remnant inorganic phase by treating the last pelleted solid material residue with a strong acid. Detailed methods are included in Additional file 1.

### Liquid chromatography-mass spectrometry analysis and protein identification

Protein samples were prepared for LC–MS by reducing and alkylating cysteines. The protein sample was precipitated by a chloroform–methanol extraction, air-dried and digested with trypsin at 37 °C overnight.

The samples were then run on a Dionex U3000 nano-flow system coupled to a Thermo Fusion mass spectrometer. Each sample was subjected to a 240-minute chromatographic method employing a gradient from 2 to 25% acetonitrile in 0.1% formic acid (ACN/FA) over the course of 200 min, a gradient to 50% ACN/FA for an additional 20 min, a step to 90% ACN/FA for 10 min, and a 10-minute re-equilibration into 2% ACN/FA in a “trap-and-load” configuration. The trap column was an Acclaim C18 PepMap100, 5 μm, 100A and the column was an Acclaim PepMap RSLC 75 μm × 15 cm (Thermo Fisher Dionex, Sunnyvale, CA). The entire run was 0.3 μl/min flow rate and the sample was ionized through a Thermo Nanospray Flex Ion Source. MS1 scans were performed in the Orbitrap utilizing a resolution of 240,000 and data dependent MS2 scans were performed in the Orbitrap by means of High Energy Collision Dissociation (HCD) of 30% using a resolution of 30,000. This was repeated for a total of three technical replicates.

Data analyses were performed using Proteome Discoverer 2.2 with SEQUEST HT scoring. Proteome Discoverer 2.2 data output was provided in an Excel format and these files can be accessed in the Additional file 2. The data base used was Homo sapiens (SwissProt TaxID = 9606, version 2017-10-25) and contained 42,252 entries. Static modification included carbamidomethyl on cysteines (= 57.021), and dynamic modification of oxidation of methionine (= 15.9949). Parent ion tolerance was 10 ppm, fragment mass tolerance was 0.02 Da, and the maximum number of missed trypsin cleavages was set to 2. Only high scoring peptides were considered with a false discovery rate (FDR) of less than 1%. An example of our mass spectrometry proteomics data set has been deposited to the ProteomeXchange Consortium via the PRIDE partner repository with the dataset identifier PXD012422 and 10.6019/pxd012422 [5].

### Light microscopy (LM), transmission and scanning electron microscopy (TEM & SEM)

In both microscopy studies (LM and EM), we followed all procedural steps recommended by the respective specialized laboratories. The observation and interpretation from LM slides and EM electron diffraction images were analyzed and discussed in agreement with the corresponding lab personnel. The LM was performed in the Morphology and Imaging Core of the LSUHSC School of Medicine (New Orleans, LA). The TEM and SEM were supported by the LSU Shared Instrumentation Facility (Baton Rouge, LA).

### Statistical and biological interpretation procedure

To elucidate and characterize the functional interactions among the proteins identified, we applied a statistical method for analyzing these complex interactions. The initial step was to validate the Proteome Discoverer data output from the LC–MS by means of characteristic quality parameters provided by the instrument. These parameters included the sum of the PEP score, coverage number (%), peptide spectrum matches (PSM), and calculated pI. The PSM were also important for quantifying (indirectly) the proteins abundance in each sample. PSM are classically used in label-free quantitative proteomics (spectral counting), and in our study, they were used in a similar way. In addition, the “calculated pI” value (pI) (although theoretical) helped validate the protein extraction method by ensuring that all pIs were represented. All samples show a pI coverage range between a minimum pH 3.8 to a maximum pH 11.8. This analysis established a measure of homogeneity.

A pairwise collinearity test of functional similarity indicators was developed to determine which of the controls could be the optimal standard control. This was accomplished statistically through successive pairwise comparisons among the proteins by a Venn analysis (L7).^7^ The procedure consisted of comparing all of the proteins from the study material to the proteins from each of the selected controls. Data structure evaluation was accomplished by using parametric and multivariate descriptive statistics (Minitab 15, student version) to determine the population data [6].

### Bio-functional evaluation

The functional evaluation of the sample proteins was accomplished through both pairwise (MB-Tt, MB-PT, and Tt-PT) and three-way (MB-Tt-PT) comparisons with all of the proteins from the controls. The functional significance of the proteins from each control was first established using G.O. and KEGG functional components from the publicly available STRING database [7, 8]. G.O. comprised three functional classification domains: Biological processes (BP), molecular functions (MF) and cellular components (CC) and their corresponding functional indicators. KEGG pathways, on the other hand, serve a complementary role and provide supplementary information on canonical pathways, diseases and functional systems.

Further systematization of the data was then achieved by an ontological filtering (ranking) scheme we developed specifically for this study. The filtering methodology consisted of taking the three most important functional indicators from each G.O. and KEEG domain and ranking each of them from the most to least relevant. The relevance was primarily determined by the p-value from the STRING analysis, or the *False Discovery Rate ґ* (*FDR)* or *Calculated Probability Value*, assuming the proteins differed from the null hypothesis. We also tabulated the number of nodes (or proteins) and the number of processes (i.e., the total number of functional indicators found at the corresponding set of nodes and added them to the functional indicator ranking). Next, the biological structure of the domains was characterized using the PANTHER functional classification system [9], a public database.

## Results

### Sample and control protein data processing and statistical analysis

Because very little is known about the mechanism(s) underlying protein deposition during salivary stone formation, we devised a series of algorithms for analyzing the stones from 29 patients. These algorithms included careful sample acquisition, proper methodology for the selection of controls, analysis of the experimental samples, and the handling and evaluation of the raw data output.

### Descriptive statistics for control and sialolith samples

Analysis of the proteins in the three controls indicated there were 196 for bone, 93 for tooth, and 69 for periosteal tissue. For the 29 sialoliths, the total number of proteins in each sample varied from a low of 116 to a high of 418. Based upon the normalized data, which corrected for the differences in protein number, the basic descriptive statistical analysis showed a minimum to maximum of 0.02 to 0.07, mean of 0.0345, median of 0.0310, and mode between 0.025 and 0.035 on the X axis. The close proximity of the mean, median, and mode indicated that the data were normally distributed and that parametric analyses were not biased.

### Control samples: proteins identity coverage and functional analysis

#### Identity coverage

The second analytical level determined the overlap in proteins among the control samples. A venn analysis produced the number of common (shared) versus unique proteins for the two positive controls (MB and Tt) and the negative control (PT). Further, when all three controls were analyzed together there were only 18 proteins in common from a total of 356 possible proteins (standardized 5.0%), which indicated there was little to no relationship among our sialolith samples. For example, pairwise comparisons found that MB and Tt only shared 46 proteins (standardized 15.91%), whereas MB and PT only shared 34 (standardized 12.87%). Note that Tt and PT have no pairwise collinear proteins.

#### Selecting the optimal standard control

The average rate of homology (i.e., Balanced Rate) between MB and each sialolith was 0.53 (53%), whereas a comparison of Tt and PT with each sialolith only had rates of 0.34 (34%) and 0.23 (23%), respectively. This observation supported the selection of MB as the best standard for comparison, and it was subsequently confirmed by a pairwise correlations analysis of each standard control with all of the sample proteins. More specifically, the results of this analysis indicated that only MB presented a significant correlation with the sialolith samples (r = 0.81). No significant correlation was found for Tt and PT. Based upon the fact that MB had a significant homology with the sialolith samples, and simultaneously had the capacity to reflect the variability among them, MB was considered an optimal standard control to which all other experimental samples could be compared.

#### Bio-functional evaluation from control proteins

The proteins from each control sample were analyzed further using the G.O.-KEGG functional STRING protocol and the criteria described previously. This analysis proved that although differentially ranked at an individual level, MB and Tt presented the same functional indicators in all domains. However, the predominant functional indicators were EE (extracellular exosomes) and BM (blood micro particles) from the Cellular Component domain. In contrast, PT differed in many functional indicators mostly related to muscle activity and transport in blood.

The functional indicators characteristic of the pooled proteins (pairwise and three way) were also determined. The proteins for the MB-Tt combination had similar functional indicator activity, but non preeminent functional indicator present at any domain. The pooled proteins for the MB-PT comparison had mixed functional indicators with higher p-values, which was an indication of a weak interaction. The Tt and PT comparison yielded a reduced number of functional indicators and weak protein interactions. The proteins pooled for the three-way MB-Tt-PT comparison had a similarly low number of indicators and relaxed interactions (much higher p-values than the other combination). The full data matrix from this analysis is shown in Additional file 3.

To further proceed with the ontological analysis, three more relevant functional indicators from each functional domain and control sample were pooled together and ranked. The data output is seen in Table 1. Interestingly, pairwise comparisons between the MB and Tt controls indicated that although the datasets have a different internal ranking, they shared 75% of the functional indicators. In addition, they shared four of the seven top indicators from both groups (highlighted in blue). These indicators belonged principally to cell component fractions containing (EE), (BM), Regulators of biological activity (RBA) and Response to stimulus (RS)). Consequently, as shown in Table 1, MB has the lowest *FDR* across functional indicators, and confirms the capacity of MB to serve as a standard control for comparing sialoliths.

**Table 1:**
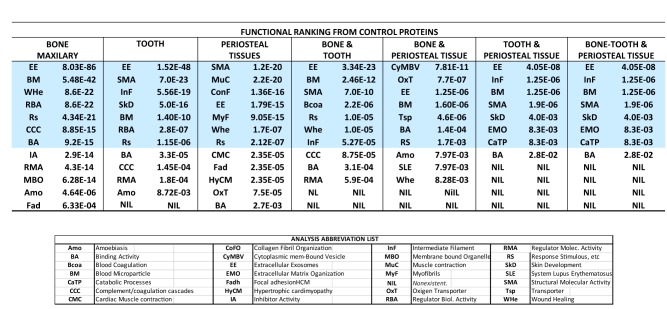
Ranked functional indicators and the abbreviations list

*Amo* Amoebiasis, *BA* binding activity, *Bcoa* blood coagulation, *CaTP* catabolic processes, *CCC* complement/coagulation cascades, *CMC* cardiac Muscle contraction, *CoFO* collagen fibril organization, *CyMBV* cytoplasmic mem-Bound Vesicle, *EE* extra cellular exosomes, *EMO* extra cellular matrix organisation, *Fadh* focal adhesion cardimyopathy, *IA* inhibitor activity, *InF* intermediate filament, *MBO* membrane bound organelle, *MuC* muscle contraction, *NIL* nonexistent, *OxT* oxigen transporter, *RBA* regulator biol. activity, *RMA* regulator molec. activity, *Rs* response stimulous, etc., *SkD* skin development, *SLE* system lupus erythematosus, *SMA* structural molecular activity, *Tsp* transporter, *Whe* wound healing

#### Sialolith samples: protein identity coverage and functional analysis

Characterization of the salivary stone samples was carried out in the same manner as the characterization of the control samples. Validation of the raw data prior to the descriptive statistics confirmed a non-biased normal distribution (data not shown) from the original data population. To assess the importance of the shared proteins among the salivary stones, a pairwise (all against all) Venn comparison was performed and the calculated protein homology coefficients (normalized) were tabulated. Based on the descriptive statistics obtained for this data matrix the mean (0.62 homology), median (0.59 homology) and mode (0.575) had similar values, with the mode located between the mean and median. From these results, we could assume that the population data were normally distributed. This assumption was confirmed using a Kormogolov–Smirnof test for normality. The test being a null hypothesis test, yielded a p-value > 0.150 (higher than 0.05 limit), and consequently the normality distribution was verified.

#### Bio-functional characterization of sialolith samples

Prior to the biological characterization of the sialolith proteins, all of the proteins from the sialolith samples were placed into two categories, either in-common (homologous) or not-in-common (non-homologous) and compared with MB proteins as discriminant factors (see Table 2a, b). Table 2a shows the dichotomy of homology and the normalized homology coefficients. In Table 2b, the descriptive statistics for the coefficients are presented. The data are characterized a Median (***Χ***) of 53, a Range (***R****)* of 30–71%, a standard deviation of (***σ***) 0.10, a (***CV*%***)* of 18.09% and a Confidence Interval (***CI***) of 0.30–0.73. The histogram in Table 2b depicts a double Mode covering homology frequency between 0.59 and 0.69 representing 62% of the data.

**Table 2:**
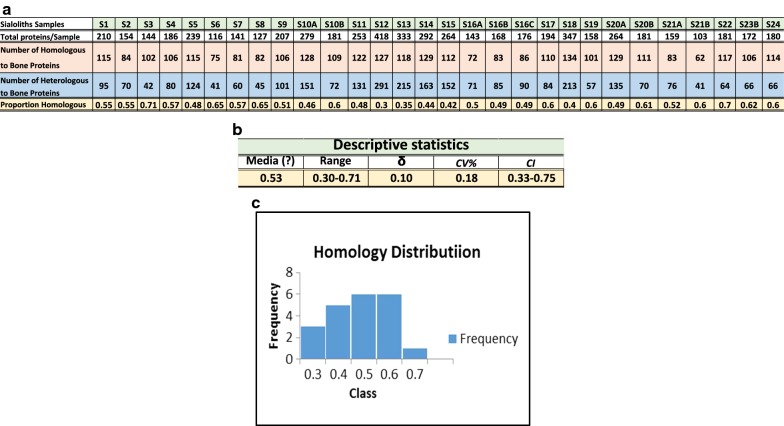
Discrimination between homologous and non-homologous proteins from maxillary bone across sialolith samples (a), basic statistics (b) and degree of homology distribution histogram (c)

#### Homologous to bone control protein group

To understand the underlying structures from the homologous to bone proteins group present across all stone samples an ontological comparison based upon functional domains and its indicators was performed. Using the same criteria applied in the analysis of control samples, we were able to identify the three highest ranked functional indicators (lower *FDR* p-value) belonging to the GO and KEGG domains (Biological Processes, Molecular Functions, Cellular Components and KEGG Pathways). More specifically, we found that Extracellular Exosomes (EE) (Average p-value of 3.8E−61) and Blood Microparticles (BM) were the most significant indicator of the Cellular-Components domain (Average p-value of 4.15E−31: as highly representative functional indicators. For additional information about the results refer to Additional file 4.

#### Non-homologous to bone control proteins group

A similar methodology was also used with the non-homologous protein group. In this case the results were similar to the homologous group. Once again the relevant functional domain was the Cellular Components and the same functional indicators, namely, Extracellular (EE) Exosomes and Blood Microparticles (BM). The differences were only that both functional indicators had higher p-values, the average p-value was 3.18E−40 for EE and 3.31E−11 for BM. The complete analysis can be accessed in Additional file 5.

#### Functional classification from functional domains and its indicators

The final objective of the functional classification was to identify the relevant biological processes and its functional subcategories belonging to of the identified functional indicators. This first step toward this objective was to assemble and categorize the functional indicators from all G.O.-KEGG domains as shown in Table 3a, b. Table 3a shows the top three functional indicators (p-values) in each domain from homologous and non-homologous stone proteins. Subsequently, the indicators from each protein identity group (homologous and non-homologous) were re-ranked according to the *RFD* data. Table 3b shows the general rank order of the functional indicators from the homologous and non-homologous groups.

**Table 3:** G.O.-KEGG bio-functional analysis (A) Rranking of G.O.-KEGG functional domains and indicators from homologous (a. 1) and non-homologous (a. 2) proteins and (B) G.O.-KEGG ranking from pooled homologous (b. 1) and non-homologous (b. 2) functional indicators set

A											
(a. 1) Homologous G.O.- kegg functional domains and tis indicators											
Biological processes			Molecular functions			Cellular components			KEGG phatways		
Rank	Confidence values		Rank	Confidence values		Rank	Confidence values		Rank	Confidence values	
Order	Min	Max	Order	Min	Max	Order	Min	Max	Order	Min	Max
Rs	2.0E–19	9.3E–08	IA	4.3E–15	2.7E–09	EE	7.2E–78	2.2E–36	CCC	9.2E–11	2.8E–02
ISP	2.6E–18	1.1E–08	BA	3.7E–19	2.6E–03	BM	9.5E–47	3.7E–06	SS	2.0E–05	2.9E–02
RA	9.6E–17	6.4E–08	SMA	3.1E–11	4.3E–05	CyMBV	3.5E–24	2.1E–05	Amo	2.3E–04	4.0E–02

(a. 2) No Homologous G.O.-KEGG functional domains and its indicators											
Rs	1.1E–15	2.3E–04	IA	1.1E–13	1.6E–04	EE	2.0E–123	1.9E–21	CCC	5.3E–15	5.2E–04
RBa	6.8E–10	3.7E–04	BA	1.9E–11	9.9E–03	MBO	5.5E–43	1.7E–06	Amo/LyS	Δ	Δ
PtID/Rs	2.4E–08	2.7E–02	RMA	1.3E–09	6.9E–03	BM	2.3E–19	1.3E–03	SS	3.3E–05	6.7E–02

B					
(b. 1) Homologous to bone			(b. 2) No Homologous to bone		
Functional indicators ranking			Functional indicators ranking		
Indicator	Max.	Min.	Indicator	Max.	Min.
(b) Homologous to bone					
EE	7.21E–78	2.2E–36	EE	1.95E–123	1.9E–21
BM	9.47E–47	3.67E–06	MBO	5.5E–43	1.70E–06
CyMBV	3.5E–24	2.06E–05	BM	2.3E–19	1.25E–03
Rs	2.0E–19	9.30E–08	Rs	1.1E–15	2.30E–04
BA	3.7E–19	2.59E–03	CCC	5.3E–15	5.20E–04
ISP	2.6E–18	1.10E–08	IA	1.1E–13	1.60E–04
RBA	9.6E–17	6.40E–08	BA	1.9E–11	9.94E–03
IA	4.3E–15	2.70E–09	RMA	1.6E–10	6.90E–03
SMA	3.13-11	2.80E–02	RBA	6.8E–10	3.70E–04
CCC	9.2E–11	4.30E–05	ptID/Rs	6.8E–10	2.66E–02
SS	2.00E–05	2.88E–02	SS	3.25E–05	6.70E–02
Amo	2.30E–04	3.96E–02	Amo/Lys	∆	∆

Subsequently we established which ranked indicators from the homologous and non-homologous sets were common and which were unique. The comparisons between the functional indicators from the two sets yielded 75% similarity. The indicators were EE (Extra Cellular Exosomes), BM (Blood Microparticles), Rs (Response to Stimulus), RBA (Regulatory Biol. Activity), IA (Inhibitory Activity), CCC (Complement and Coagulation Cascade), SS (Salivary Secretion), and Amo (Amoebiasis). The other 25% of the indicators were of dissimilar nature. In this case, CyMBV (Cytoplasmic Membrane Bound Vesicles), ISP (Immune System Processes) and SMA (Structural Molecular Activity) were unique from the homologous group MBO (Membrane Bound Organelles), RMA (Regulatory Molecular Activity), and PtlD (Platelet Deregulation) were unique from the BM group.

Thus, the highly ranked EE and BM functional indicators were selected as the common representatives from homologous and non-homologous groups. The groups were also annotated as EE^h^ for the homologous and EE^nh^ for the non-homologous. The same criteria applied for BM^h^ and BM^nh^, respectively. This designation permitted us to find the most representative sialolith sample from homologous and non-homologous groups containing the most relevant/representative set of proteins, which in turn was used to classify the functional indicators that characterize salivary stones via PANTHER bio-informatics platform [10, 11]. So, for EE^h^, sample S20A was identified as containing the most representative and relevant set (of proteins) with a p-value of 7.2E−78. And for EE^nh^, sample S18 contained the most representative and relevant set with a p-value of 2.0E−123. The same criteria were applied with respect to BM. For BM^h^, sample S22 had a set with a p-value of 9.5E−47, and for BM^nh^, sample S24 had a set with a p-value of 2.3E−19. The Panther classification platform was then used to identify the biological processes and its sublevels from the selected sialoliths samples.

The results from PANTHER are shown in Table 4a (I and II). The Panther scores were Table 4a (I) subjected to basic statistical analysis to determine their normal distribution and other descriptive statistical estimators. The data were normally distributed with narrow variance around the mean (data not shown). The correlations from the four indicators EE^h^, EE^nh^, BM^h^, and BM^nh^ were then compared in a pairwise manner. Each combination had a high correlation coefficient, with an average ***r***-value of 0.92. The PANTHER scores for the selected sialolith samples for all four indicators were all in agreement. Thus, the best (higher) Panther scores belonged to specific categories from biological processes such as cellular process, response to stimulus, and metabolic process. Two proteins of interest in EE^nh^, TAGLN-2 (ID P37802-2) and AMBP (ID P02760) were found during manual curation. TAGLN-2 (ID P37802-2) is a protein with an unknown function and present in 12.5% of the sialoliths samples, whereas AMBP (ID P02760) is an inhibitory protein that among other functions inhibits the crystallization of calcium oxalates and present in 54% of the sialoliths samples.

**Table 4:** (a) Biological processes categories of Shared (I) and Unique (II) functional indicators identified by PANTHER classification system, (b) Subcategories of the main biological processes

(a) Biological processes categories of Shared (I) and Unique (II) functional indicators identified by PANTHER classification system										
Biological process	(I) Shared functional indicators				(II)Panther scores for unique indicators^a^					
	Homologous		Non homologous		Homologous			Non nomologous		
Categories	EE^h^	EE^nh^	BM^h^	BM^nh^	CyMBV	ISP	SMA	MBO	RMA	PtID
Biological adhesion	0.02	0.02	0.02	0.01	0.01	0.02	0.02	0.02	0.02	0.01
Biological regulation	0.12	0.13	0.13	0.13	0.11	0.11	0.13	0.09	0.12	0.13
Cell. comp. organiz/bio gen.	0.13	0.14	0.09	0.07	0.11	0.08	0.12	0.10	0.13	0.07
Cellular process	0.46	0.34	0.33	0.31	0.30	0.39	0.38	0.39	0.32	0.31
Developmental process	0.02	0.03	0.01	0.02	0.01	0.01	0.03	0.02	0.03	0.02
Immune system process	0.15	0.14	0.20	0.19	0.16	0.19	0.12	0.21	0.12	0.19
Localization	0.10	0.10	0.09	0.10	0.08	0.09	0.13	0.11	0.10	0.10
Metabolic process	0.28	0.24	0.24	0.26	0.27	0.32	0.26	0.28	0.26	0.26
Multicell. organismal process	0.03	0.03	0.03	0.02	0.03	0.02	0.02	0.02	0.02	0.02
Reproduction	0.00	0.00	0.00	0.00	0.00	0.00	0.00	0.00	0.00	0.00
Response to stimulus	0.32	0.31	0.35	0.32	0.31	0.36	0.30	0.38	0.28	0.32

(b) Main subcategories from relevant biological process		
Cellular process	Response to stimulus	Metabolic process
Cellular metabolic process	Cellular response to stimulus	Cellular metabolic process
Cellular membrane organization	Immune response	Primary metabolic process
Cellular response to stimulus	Response to stress	Organic metabolic process
Signal transduction	Response to chemicals	
Cell cycle	Response to abiotic stimulus	

*h* homologous *nh* no-homologous

^a^Panther scores: are the percent of gene hits against the total function hits for each indicator

We next addressed the unique or dissimilar functional indicators (25%) from both groups seen in Table 4a (II). The unique functional indicators selected from the homologous set were CyMBV (cytoplasmic membrane-bound vesicles), ISP (immune system processes), and SMA (structural molecular activity) and they were drawn from S19, S17, and S13, respectively. From the non-homologous group, the functional indicators identified were MBO (membrane-bound organelles), RMA (regulatory molecular activity), and PtlD (platelet degranulation) and they were drawn from S9, S14, and S24, respectively. The normalized data generated by PANTHER were also statistically analyzed satisfying its normal distribution.

The Panther classification system was also able to identify the subcategories for the three Biological processes (i.e., cellular process, response to stimulus, and metabolic process) that scored the highest for both the homologous and non-homologous proteins (Table 4b). The rest of the bio-indicators were evenly distributed. As the proteins of these indicators bore little relationship to mineralogical deposition, they were not investigated further.

#### Light microscopy (LM), transmission electron microscopy (TEM), and scanning electron microscopy (SEM) of sialolith samples

**Fig. 2:**
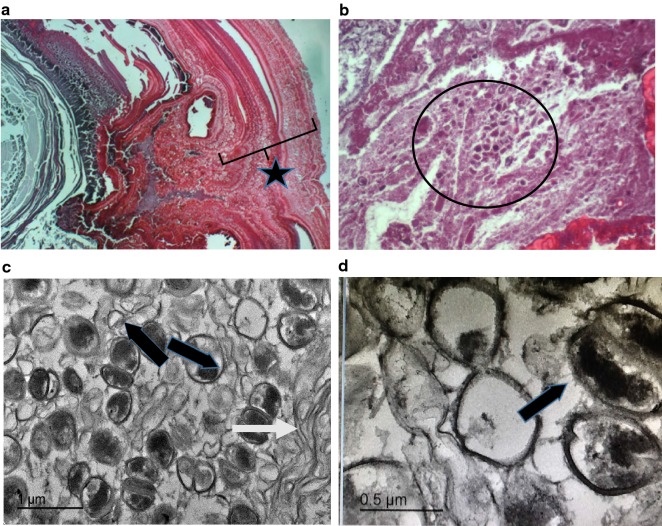
Light microscopy and electron microscopy analysis from sialoliths samples; light microscopy (lm) 4× magnification **a** concentric laminae (star) and 6× magnification **b** globular structures in external laminae (circle). Transmission Electron Microscopy (TEM) 1 μm scale **c**. Intra-vesicular and extra-vesicular deposition of inorganic matter (black arrows) and large membranous bodies (white arrow) **d** details of extra and intra vesicular inorganic deposition (arrow) at 0.5 ηm scale

**Fig. 3:**
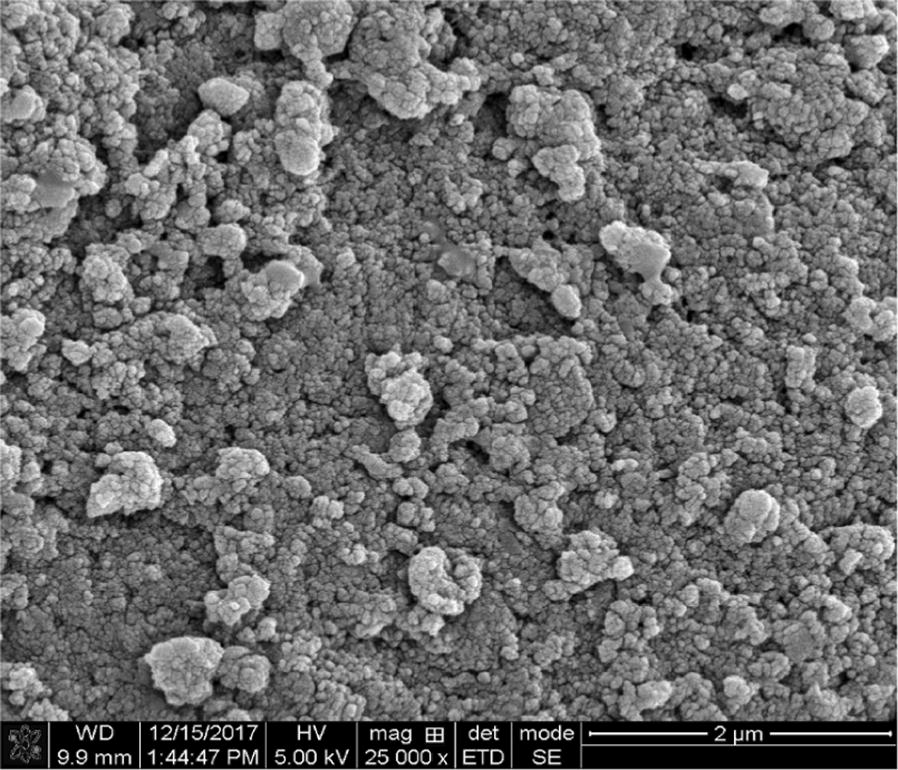
Scanning electron microscopy image from an internal stone laminae showing the globular tridimensional structure on a 2 μm scale

To investigate further the possible relationship between exosomes and salivary stone formation, we used a wide range of microscopy studies. LM results are shown in Fig. 2, plates a–d. Figure 2, plate (a), represents a low magnification image of a sialolith sample. This image illustrates the irregular concentric laminar structure of the stones, with hollow spaces interposed (star). At higher magnification (Fig. 2 plate (b)), the globular structures that form part of the external lamellas can be seen (circle). In the TEM analysis (Fig. 2, plates (c) and (d)), spherical corpuscles (vesicles) of around 0.5 to 2 µm in diameter are shown. The size of some these micro vesicles correspond to well-established exosomal dimensions and is suggestive of their presence in the salivary stone. Note also the large number of exosomal-like features in the image (Fig. 2c, d) that appear to have internal and surface opaque contrast areas, which could be an indication of deposition of microcrystalline inorganic compounds (arrows). The SEM image in Fig. 3 provides a three dimensional view of the structure of salivary stones. It can be inferred from this SEM image that individual exosomes tend to coalesce in primary globules that, in turn, assemble together forming secondary and tertiary structures. Similar structures have also been observed in kidney stones.

## Discussion

### Sialolithiasis concerns

The clinical problem of sialolithiasis and its subsequent effects on morbidity and quality of life mandate continued efforts at improving treatment options and strategies. Indeed, gland preservation and stone management have been improved with endoscopic and combined open surgical techniques, but treatments are still limited by stone position, hardness, and interaction with the salivary ductal system or gland. These limitations are often tied to our lack of understanding of both the formation of stones, and the interrelationship between the protein matrix and inorganic components of the stones. The latter could be vital to developing better strategies for stone management by allowing for partial or total dissolution, and consequently, gland preservation through a minimally invasive intervention.

Although there is an abundance of data about nephrolithiasis, there is limited data on sialolithiasis; in addition, the current research on sialolith composition is contradictory in some cases. A case in point was our inability to find agreement in previous research on the inorganic composition of sialoliths. For instance, Taher [12] studied stones from 95 patients and found the composition to be 89.8% phosphate salts (hydroxyapatite), 7.9% oxalates, and 2.3% urate salts. In contrast, Kasaboğlu et al. [13] sampled six patients in which all of the stones contained large amounts of calcium phosphate salts (mostly hydroxyapatite) and traces of Mg, K, Na, Cl, Al and Fe. A very recent study by Stelmach et al. [14], which was based upon 46 patients, reported the presence of C, Ca, O, P, and S. Although the author did not calculate any molar proportions to estimate the potential mineral composition, they proposed the main components were phosphate salts (hydroxyapatite). Grases et al. [15] introduced other interesting aspects in the mineralogical development of salivary stones. They found that saliva contains the crystallization inhibitor phytate, also known as myo-inositol hexaphosphate, which is an important etiological factor in sialolith development. In addition, Gryčova et al. [16] presented evidence that sialoliths contain various metals like Pb, Ti, and Zn.

To determine the inorganic composition in vesicular structures forming the stone that are possibly the foundational microcrystals of sialoliths, we utilized a novel chemical imaging technique using laser-ablation for detecting the mineralogical composition of the stones. Although our sample size was smaller than many in the literature, we found consistent amounts of Ca, C, O, P, Mg, S and traces of I, Ti, Zn and Al in the salivary stones. Based upon the molar proportions of Ca, C, O, P, and Mg present, we could estimate similar proportions of calcium phosphates and calcium oxalates. Another interesting finding was that the fraction of Mg was higher than expected, suggesting that the mineralogical compound Struvite could be present too. Together, our findings along with those in the literature indicate that further investigation using refined techniques will be required to elucidate the chemical composition of salivary stones.

### Protein matrix vs organic phase: current evidence

Previous research regarding the organic phase of sialoliths was also quite variable. In a study by Osuoji et al. [17], for example, they found that only 5% of the organic phase was soluble in water after demineralization. The protein content consisted of seventeen amino acids, and this same proportionality occurred across samples. There were also no characteristic amino acids for collagen and keratin (hydroxyproline and cystine). The carbohydrate content in salivary duct stones was demonstrated to be small, with glucose and mannose as the major components. The lipid fraction was also observed to have phospholipids, cholesterol, cholesterol esters, fatty acids (the large component), and di- and triglycerides. Teymoortash et al. [18] analyzed sialoliths from Wharton’s duct (a duct of the submandibular salivary gland) and discovered that the organic materials were predominantly concentrated in the outer shell of the stones and their components were glycoproteins, mucopolysaccharides, lipids, and cellular detritus (Phospholipids). Considerable research carried out by several groups such as Sabot et al. in 2012 [19]; Szalma et al. 2012 [20]; Faklaris et al. 2013 [21]; and Kraaji et al. 2014 [22]) have also advanced our understanding of stone architecture by showing that some can have a pure protein nucleus surrounded by mixed organic and carbonate apatite layers; whereas others can have internal layers of apatite covered by a dense and varnished crust of proteins and other organic compounds. In addition, Yiu et al. [23] and Ho et al. [24] recently reported that bone forming mechanisms involved in the early stages of kidney stone development and arterial calcification also require the participation of proteins and transcription factors.

### The discriminant standard

Using our proteomics approach to analyze the 29 stone samples, 824 unique proteins were identified from the 6934 detected. As with any large data set, the analysis and distillation of useful information was challenging. Therefore, we utilized a novel methodology for (1) identifying a discriminant standard to dichotomously separate the sialolith samples, (2) categorizing the functional domains and indicators of these dichotomous groups via STRING analysis, and (3) classifying the selected indicators using PANTHER algorithms (Fig. 1). This method also took advantage of the basic principles underlying classical population genetics by capturing the population’s variability and contrasting it with a standard control, which then allowed us to identify the most explanatory and meaningful data. This methodology was clearly reliant on establishing controls that could serve as appropriate comparisons for all of the stones. In this case, we compared sialolithiasis with the mineralogical deposition mechanisms that form bone and teeth as positive controls (MB and Tt), and a tissue absent mineralogical deposition as a single negative control (PT). Among these, MB fulfilled the requirements of an optimal standard control, because it had an average of 53% homology across samples and this homology was highly correlated (***r ***= 0.8) with the total proteins characterizing the sialoliths.

### Protein content of salivary stones and their functional significance

As the preferred standard, MB allowed for the division of each sialolith into two protein groups, one having common proteins with bone, the homologous group, and one having no common proteins with bone, the non-homologous group (see Table 2). Both the homologous and non-homologous were subjected to a stringent computational analysis of biological function domains and products (G.O. and KEGG) followed by the ranking of functional indicators from each biological domain. These steps were fundamental in obtaining the optimal protein set to proceed with the functional classification, so that the top functional indicators in both groups could be identified—namely, EE and BM (see Table 4a, b). As a result, we were also able to demonstrate that the EE subgroup was more significant than BM, and that EE were the principal carriers of elements from primary metabolic processes and immune reactions via large amounts of acute phase reactants (APR), and to a lesser extent, components of cellular organization and transport. In turn, this strategy identified the AMBP protein responsible for the solubility of calcium oxalates that may be of critical importance for stone formation. AMBP inhibits the crystallization of calcium oxalates and was present in 54% of the sialoliths. Notably, calcium oxalates are the main inorganic component of salivary stones and other bodily concretions.

### Potential relevance of extracellular exosomes, blood microparticles, and other membranous structures

Previously published literature has shown that exosomes can have different subtypes and carry characteristic cargo elements as demonstrated by Willms et al. [25]. Our study also revealed the potential role for EE in the structure of sialoliths. It also highlighted the role that EE have as carriers/transporters of proteins from the immunologic and metabolic processes and their regulators. They are a constitutive part of the extracellular matrix and apparently a site for deposition of amorphic mineral microcrystals. We believe they form tridimensional globular structures giving salivary stones a variable organization and texture, as shown by the TEM and SEM analysis. Interestingly, our microscopy studies also uncovered large membranous structures resembling collapsed blood components like lymphocytes (Fig. 2 plates, c and d), which suggests immunological constituents involvement as proposed by DiGiuseppe [26].

During the collection of the stones, special care was taken to eliminate the saliva and blood contamination by meticulous and repetitive decontamination and cleansing procedures described in the materials and methods. In spite of this protocol, abundant saliva and blood proteins were identified by the subsequent MS analysis. Consequently, there is the strong possibility that these proteins are systematically deposited during the stone’s formation. The possibility that saliva is a source for exosome is also supported by the work of Shapiro et al. [27] and Han et al. [28].

The role of EE could have more intricate implications in stone formation as well. For example, Kapsogeourgou et al. [29] working with salivary gland epithelial cells found that they also constitutively secrete exosomes carrying major autoantigens such as anti-ribonucleoproteins antigens (RNP). Our study found that functional indicators from the biological processes domains such as immune system processes (ISP) and immune and defense responses (Rs) had highly significant FRD p-values and were an integral part of the extracellular matrix. These results support the possibility that these proteins may be deposited in salivary stones during stone growth, and therefore, could have multiple exosomal origins (Additional files 4, 5).

### Inorganic composition

The mineralogical composition of the salivary stones in our study resembled that of kidney stones (nephrolithiasis) produced by hyperoxaluria; a process indicated by accumulation and super saturation of calcium oxalates (CaOx) in urine. According to Sriram et al. [30], the etiology of primary hyperoxaluria can be divided into two autosomal recessive disorders of the endogenous oxalate pathway. The type-I disorder, PH I (AGXT1), is characterized by a functional defect of the hepatic enzyme alanine:glyoxylate amino transferase, whereas the type-II disorder, PH II (GRHPR), is characterized by a deficiency of glyoxylate:hydroxypyruvate reductase, leading to oxalate and glyoxylate accumulation [31]. Under homeostatic cell conditions, this metabolic pathway is responsible for transforming toxic oxalates to glycine and then eliminating it via urine or processing it in the liver. Sriram et al. [30] also stated that AGXT1 and GRHPR normally control oxalate, but no traces of these enzymes were found in our protein samples. However, coincident with many reports in the nephrolithiasis literature, osteopontin (OPN), bikunin (BK), heparan sulfate (HS), and prostaglandins (PG) were all detected. All of them are well known mediators of inflammatory processes and extracellular matrix production. In addition, angiotensin and proteins belonging to the renin-angiotensin system were found (e.g., Cathepsin G, Kallikrein, Lysosomal Pro-X carboxypeptidase, aminopeptidase N), which via NADPH-oxidase and reactive oxygen species (ROS) activate P38, MAPK and JUNK. These mediators, in turn, increase the expression of OPN, BK, HS and PG among others [32–34].

## Conclusions

We isolated and identified the protein fractions from 29 sialoliths using an LC–MS workflow. The subsequent proteomic and bioinformatic analysis was effective in revealing the complexity of the protein data obtained and creating a smaller more informative subset of sample proteins. The analysis also revealed that two important possibilities exist in the formation of sialoliths: (1) the exosomal APR content (evidence of immune activation) and the presence of lymphocytic structures, and (2) the mechanistic similarities between the formation of salivary and kidney stones, and the potential relationship with hyperoxaluria. These similarities further support a hypothesis that all pathological bodily concretions like glandular stromal stones (salivary, thyroid, lung, heart, pineal etc.) may share a general common formational pathway. Elucidating such a mechanism could potentially influence research methodology, device and technology development, and clinical management of lithiasis in general. Future studies will emphasize quantitative inorganic analyses, and thus, unequivocally determine the contribution of the mineralogical composition in the stone formation. They also will help to identify the origin of the exosomal influence in the formation of sialoliths.

## Supplementary information

**Additional file 1.** Protein extraction protocol.

**Additional file 2.** Raw mass spectrometry data output.

**Additional file 3.** G.O.-KEGG analysis of control samples (Max. bone, tooth and periosteal tissue proteins).

**Additional file 4.** G.O.-KEGG analysis of sialoliths homologous to bone proteins.

**Additional file 5.** G.O.-KEGG analysis of sialoliths non- homologous to bone proteins.

## Data Availability

All data generated or analyzed during this study are included in the published article in supplementary files. Representative raw data generated or analyzed during the project are available in the repository (link)

## Abbreviations

ACN/FA: Acetonitrile/formic acid
Amo: Amoebiasis
BM: Blood microparticles
BP: Biological processes
CC: Cellular components
CCC: Complement and coagulation cascade
CyMBV: Cytoplasmic membrane bound vesicles)
EE: Extracellular exosomes
EM: Electron microscopy
FDR: False discovery rate
G.O: Gene ontology
HCD: High energy collision dissociation
IA: Inhibitory activity
ISP: Immune system processes
KEGG: Kyoto encyclopedia of genes and genomes
LC–MS: Liquid chromatography mass spectrometry
LM: Light microscopy
MB: Maxillary bone
MBO: Membrane bound organelles
MF: Molecular functions
PEP: Posterior error probability
pI: Calculated isoelectric point
PSM: Peptide spectrum matches
PT: Maxillary periosteal tissue
PtlD: Platelet deregulation
RBA: Regulators of biological activity
RIPA: Radio-immunoprecipitation assay buffer
RMA: Regulatory molecular activity
RS: Response to stimulus
SEM: Scanning electron microscopy
SMA: Structural molecular activity
SS: Salivary secretion
TEM: Transmission electron microscopy
Tt: Tooth

## Footnotes

1 L1: CostHelper- Health & Personal Care: http://health.costhelper.com/kidney-stone.html.

2 L2: Right Diagnosis Statistics about urinary stones: http://www.rightdiagnosis.com/u/urinary_stones/stats.htm.

3 L3: UpToDate^®^: http://www.uptodate.com/contents/epidemiology-of-and-risk-factors-for-gallstones.htm.

4 L4: Clinical Consult: http://www.unboundmedicine.com/5minute/view/5-Minute-Clinical-Consult/116128/all/Choledocholithiasis.htm.

5 L5: https://en.wikipedia.org/wiki/Sialolithiasis.

6 L6: The Statistics Portal: http://www.statista.com/topics/1764/global-pharmaceutical-industry/.htm.

7 L7: http://bioinformatics.psb.ugent.be/webtools/Venn/.
