# Supplementary material for: A comprehensive analysis of sialolith proteins and the clinical implications
Source: Clin Proteomics. 2020 Mar 31;17:12. doi: 10.1186/s12014-020-09275-w (PMC7110646; doi:10.1186/s12014-020-09275-w)
Supplement: Supplementary file 1 — Additional file 1. Protein extraction protocol. [file 12014_2020_9275_MOESM1_ESM.docx]

**Additional File 1: Protein Extraction Protocol**

**Sequential steps and procedures:**

**Protein Extraction Procedure:**

Protein extractions techniques commonly used in literature were reviewed and studied. Based on this review, we selected a modification of the bone extraction protein method used for bone proteomic analysis developed by Xiaogang Jiang et al 2007 (4). This extraction procedure has four sequential steps beginning with the stone’s maceration with liquid nitrogen, followed by a demineralization step, and two consecutive treatments of pelleted macerates with Guanidine and RIPA buffers respectively, and a final treatment of the last pelleted solid material residue with a strong acid for a total dissolution of the remnant inorganic phase. All supernatant containing the proteins from each step were precipitated using the acetone-trichloracetic (TCA) method. SDS-PAGE was performed to qualitatively assess the protein extracts before preparation for LC-MS acquisition and analysis

**a) *Maceration:***

The optimal amount of sialolith for protein extraction was 200 to 250 mg per sample. Stones heavier than this amount were fragmented into smaller pieces and added until the standard weight was achieved. The samples were then placed in an aluminum mortar half filled with liquid nitrogen for 2-3 minutes to stabilize them. Following stabilization, the samples were ground to a fine powder with an aluminum pestle.

**b) *Protein Extraction Steps***

1. Post-Demineralization Extraction:

The powder (macerate) from each sample was transferred to a centrifuge tube containing 2 ml/ tube of the Demineralization Solution (DS) consisting of an aqueous solution of 2.4 M HCl. The macerate was vortexed for 3 minutes and incubated for 24 hours at 4⁰ C. After incubation, the suspension was centrifuged for 15 minutes. This supernatant was transferred to a fresh tube containing an acetone-TCA precipitation solution and incubated at -20º C for at least 24 hrs. The precipitation solution was composed of 15% (w/v) trichloracetic acid (TCA), in 100% cold acetone. The protein precipitated from this solution gave rise to the first fraction *(****EXTRACTION 1)***. The remaining demineralized pellet was used for the subsequent Chaotropic procedure.

1. Chaotropic Protein Solubilization:

The demineralized pellet was resuspended by vortexing in guanidine extraction buffer and incubated at 4C⁰ for 24 hrs. Following the incubation, this chaotropic solution was centrifuged as in the previous step. The supernatant at the top was then transferred to a tube containing the acetone-TCA precipitation solution and incubated at 20º C for another 24 hrs. The precipitated proteins from this extraction constituted the second fraction *(****EXTRACTION 2****)*. The residual pellet from the chaotropic treatment was washed twice with sterile deionized water subjected to sporadic strong vortexing followed by a centrifugal pelleting.

1. Mild RIPA Protein Extraction:

The RIPA buffer was composed of a solution containing 20 mM Tris, pH 7.5, 150 mM NaCl, 2 mM EDTA, 0.05% Nonidet P-40, 0.1% SDS, 1 mM DTT, 1 mM PMSF. To begin this extraction, 2 ml of complete RIPA buffer was added to the residual pellet from the previous centrifugation and vortexed and incubated for 24 hours at 4⁰ C. As in previous extraction steps, after incubation, the suspension was centrifuged and the liquid phase transferred to a centrifuge tube containing acetone-TCA precipitation solution followed by another incubation at described conditions. The protein precipitated by this treatment was the third fraction ***(EXTRACTION 3)***.

1. Extraction with Concentrated HCL

The residual pellet from the RIPA buffer incubation was re-suspended in 1 ml of 12.1 M HCL solution, and incubated for another 24 hours at 4⁰ C. After incubation, the re-suspended mixture was centrifuged as indicated in previous steps. The liquid fraction was then transferred to a tube containing the corresponding acetone-TCA precipitation solution for the same 24 hours of incubation at -20⁰ C that was used in all of the previous steps. The coalescent protein precipitate obtained after centrifugation was the fourth fraction ***(EXTRACTION 4)***.
